# Supplementary material for: Cryo-EM architecture of a near-native stretch-sensitive membrane microdomain
Source: Nature. 2024 Jul 24;632(8025):664–71. doi: 10.1038/s41586-024-07720-6 (PMC11324527; doi:10.1038/s41586-024-07720-6)
Supplement: Supplementary file 1 — Supplementary Discussion, Supplementary Methods, References, Tables 1–5 and Supplementary Data 2–4. [file 41586_2024_7720_MOESM1_ESM.pdf]

nature portfolio

https://doi.org/10.1038/s41586-024-07720-6

Supplementary information

Cryo-EM architecture of a near-native stretch-sensitive membrane microdomain

In the format provided by the authors and unedited

Supplementary Information

CryoEM architecture of a near-native stretch-sensitive membrane microdomain.

Jennifer M. Kefauver, Markku Hakala, Luoming Zou, Josephine Alba, Javier Espadas, Maria G. Tettamanti, Jelena Gajić, Caroline Gabus, Pablo Campomanes, Leandro F. Estrozi, Nesli E. Sen, Stefano Vanni, Aurélien Roux, Ambroise Desfosses, Robbie Loewith

Table of Contents

Supplementary Discussion ***3***
Native-source eisosome isolation as tubules 3
Membrane voids represent ergosterol molecules stably localized at amphipathic helix 3
Physiological implications of eisosome lattice stretching 3
Role of amphipathic helices in mechanical stress sensing 3
Lipid organization by membrane scaffold proteins beyond the MCC/eisosome 4

Supplementary Methods ***5***
Fluorescence Recovery After Photobleaching (FRAP) data analysis 5
Lipid sorting coefficient data analysis 5

Supplementary References ***6***

Supplementary tables ***9***
Table 1. Symmetry parameters and diameters of helical structures. 9
Table 2. Lipids used in this study. 10
Table 3. Lipid mixtures used in this study. 11
Table 4. FRAP halftimes in seconds and percentages of mobile fractions. 12
Table 5. Yeast strains used in this study. 13

Source data files ***14***
Source data Fig 2.xlsx
Source data Fig 3.xlsx
Source data Fig 4.xlsx
Source data Fig Ext Data 6.xlsx
Source data Fig Ext Data 7.xlsx
Source data Fig Ext Data 8.xlsx
Source data Fig Ext Data 9.xlsx
Source data Fig Ext Data 10.xlsx

Supplementary data ***14***
Supplementary Data 1. Mass spectrometry analysis of native-source eisosome preps. (See .xlsx file)
Supplementary Data 2. NMR of ergosterol. 15
Supplementary Data 3. NMR of brominated ergosterol. 16
Supplementary Data 4. Source gel image. 17

Supplementary Discussion

Native-source eisosome isolation as tubules

While MCC/eisosomes have been shown to have a furrow-like halfpipe structure *in vivo*[1,2], our isolated eisosome tubules appear to be closed, continuous helices of Pil1/Lsp1 proteins. Similar tubular structures have previously been shown in reconstituted Pil1 samples and eisosomes purified from yeast[2]. Apparently, once the eisosomes are freed from the plasma membrane by our gentle purification methods, the Pil1/Lsp1 lattice realigns to form a helical conformation around a lower-energy tubular state of the plasma membrane lipids to which it is bound. Alternatively, the eisosomes that we have isolated were already in a tubulated state *in vivo*, a behavior that has been observed in deletion strains of the eisosome-resident Sur7-family proteins and upon palmitoylcarnitine treatment in *S. cerevisiae*[3]*,* as well as Pil1 overexpression in *S. pombe*[4,5].

Membrane voids represent ergosterol molecules stably localized at amphipathic helix

Sterols, with their rigid ring structures, exhibit reduced Coulombic potential compared with tightly packed phospholipid tails[6–8]. Examples of sterol/AH interactions have been previously observed[9–11]. Notably, MD simulations with the AH of PIP5K predict that cholesterol fills packing defects near aromatic side chains and wedges between the acyl chains of poly-unsaturated PI(4)P[12].

Physiological implications of eisosome lattice stretching

We propose that eisosome lattice stretching leads to lipid mobilization, which serves as a general mechanism to free sequestered factors to signal membrane stress. These sequestered factors need not be proteins; destabilizing interactions between the Pil1/Lsp1 lattice and sterols or PS could also initiate signaling processes. For example, our mutants predicted to be compromised in sterol coordination are hypersensitive to Nystatin, a macrolide antibiotic that binds sterols in the plasma membrane leading to cell leakage and death, suggesting that sequestration of sterol at MCC/eisosomes can also regulate the amount of free sterol in the PM. Lastly, although we have presented eisosome lattice stretching as a possible trigger leading to release of sequestered factors, other membrane perturbations that result in changes in lipid dynamics, for example, thermal shock or amphiphilic toxins, could also be sensed by this system.

Role of amphipathic helices in mechanical stress sensing

The function of AHs to organize the lipids within the membrane and connect mechanical stretching to the dynamics of those lipids may be a conserved feature of mechanosensitive proteins. In fact, AHs from many mechanosensitive ion channels, including Piezo1[13–15], Piezo2[16],

TREK/TRAAK[17,18] and OSCA1.2[19] have bulky side chains inserted to the membrane and Piezos, in particular, have been proposed to modulate and be sensitive to the lipid composition of the membranes in which they function[20–22].

Lipid organization by membrane scaffold proteins beyond the MCC/eisosome

Although the MCC/eisosomes are a fungi-specific membrane feature[23], many of the principles of lipid coordination by BAR domain proteins and other proteins that form lattices on membrane surfaces are likely to be conserved. For example, caveolae in mammalian cells, scaffolded by the interaction between caveolins (integral membrane proteins) and cavins (peripheral membrane proteins) are proposed to concentrate cholesterol, PS, and $PI(4,5)P_2$[24–26], as well as flatten in response to sterol removal from the plasma membrane[27], suggesting that they share many features with the MCC/eisosome membrane microdomain. There are also several other examples of mammalian membrane domains scaffolded by BAR-domain proteins including the t-tubules of cardiac and skeletal muscle tissue that are stabilized by BIN-1[9,28,29]. In fact, the organization and alteration of the dynamics of membrane lipids through interactions between membrane scaffolding proteins, charged lipids, and sterols could extend across a wide variety of structurally and functionally diverse proteins that have been proposed to associate with membrane microdomains, from focal adhesion proteins[30,31] to ESCRTs[32,33] to myelin-specific proteins[34,35]. Our detailed characterization of the MCC/eisosome membrane microdomain provides a novel context for understanding how protein-lipid interactions participate in cell signaling functions.

Supplementary Methods

Fluorescence Recovery After Photobleaching (FRAP) data analysis

The FRAP data was analyzed with Fiji v1.54f by measuring intensity over time from the bleached region (region-of-interest), the region on nanotube outside the photobleached region (bleaching correction) and the region outside nanotube (background). After background subtraction and bleaching correction, the data for normalized to the intensity values before photobleaching. Graphs were generated with Origin Pro 2022 v9.9.0.225 (OriginLab Corp.). To calculate recovery halftimes and mobile fractions, we used either one-phase exponential equation:

$$y = A1 \times e^{t/\tau} + y0$$

or two-phase exponential equation:

$$y = A1 \times e^{t/\tau 1} + A2 \times e^{t/\tau 2} + y0$$

where A is value of y in plateau, t is time, τ is a time constant and y0 is the value of y when t=0.

Recovery halftimes were then calculated with equation:

$$\mathrm{halftime} = \tau \times ln(2)$$

Lipid sorting coefficient data analysis

Sorting coefficients were calculated for each of the mentioned lipids using Atto 647N DOPE as the reference lipid with the following equation:

$$Sorting\ coefficient = \frac{(F_{Tested\ lipid}/F_{Atto\ 647\mathrm{N\ DOPE}})\ under\ Pil1}{(F_{Tested\ lipid}/F_{Atto\ 647N\ DOPE})\ bare\ lipid\ nanotube}$$

where $F_{Tested\ lipid}$ and $F_{Atto\ 647N\ DOPE}$ are the integrated fluorescence densities of the lipid problem and lipid reference integrated from the lipid nanotube plot profiles (Extended Data Fig 8C) after background subtraction and neglecting the polarization factor[36]. Microscopy images were visualized and data extracted using Fiji v1.54f software. Data was analysed using Origin Pro 2022 (OriginLab Corp.) and plots were generated using GraphPad Software (GraphPad Prism 10.1.1).

Supplementary References

1. Strádalová, V. *et al.* Furrow-like invaginations of the yeast plasma membrane correspond to membrane compartment of Can1. *J. Cell Sci.* **122**, 2887–2894 (2009).
2. Karotki, L. *et al.* Eisosome proteins assemble into a membrane scaffold. *J. Cell Biol.* **195**, 889–902 (2011).
3. Haase, D. *et al.* Tetraspanner-based nanodomains modulate BAR domain-induced membrane curvature. *EMBO Rep.* **24**, e57232 (2023).
4. Kabeche, R., Baldissard, S., Hammond, J., Howard, L. & Moseley, J. B. The filament-forming protein Pil1 assembles linear eisosomes in fission yeast. *Mol. Biol. Cell* **22**, 4059–4067 (2011).
5. Kabeche, R., Howard, L. & Moseley, J. B. Pil1 cytoplasmic rods contain bundles of crosslinked tubules. *Commun. Integr. Biol.* **8**, 990848 (2015).
6. Wennberg, C. L., Van Der Spoel, D. & Hub, J. S. Large influence of cholesterol on solute partitioning into lipid membranes. *J. Am. Chem. Soc.* **134**, 5351–5361 (2012).
7. Moss, F. R. *et al.* Brominated lipid probes expose structural asymmetries in constricted membranes. *Nat. Struct. Mol. Biol.* **30**, 167–175 (2023).
8. Unwin, N. Structure of a cholinergic cell membrane. *Proc. Natl. Acad. Sci. U. S. A.* **119**, (2022).
9. Daum, B. *et al.* Supramolecular organization of the human N-BAR domain in shaping the sarcolemma membrane. *J. Struct. Biol.* **194**, 375–382 (2016).
10. Martyna, A. *et al.* Cholesterol Alters the Orientation and Activity of the Influenza Virus M2 Amphipathic Helix in the Membrane. *J. Phys. Chem. B* **124**, 6738–6747 (2020).
11. Rahman, K. *et al.* Cholesterol Binds the Amphipathic Helix of IFITM3 and Regulates Antiviral Activity. *J. Mol. Biol.* **434**, 167759 (2022).
12. Nishimura, T. *et al.* Osh Proteins Control Nanoscale Lipid Organization Necessary for PI(4,5)P2 Synthesis. *Mol. Cell* **75**, 1043-1057.e8 (2019).
13. Guo, Y. R. & MacKinnon, R. Structure-based membrane dome mechanism for Piezo mechanosensitivity. *Elife* **6**, (2017).
14. Saotome, K. *et al.* Structure of the mechanically activated ion channel Piezo1. *Nature* **554**, (2018).
15. Zhao, Q. *et al.* Structure and mechanogating mechanism of the Piezo1 channel. *Nature* **554**, 487–492 (2018).
16. Wang, L. *et al.* Structure and mechanogating of the mammalian tactile channel PIEZO2. *Nature* **573**, 225–229 (2019).

17. Milac, A. L. *et al.* Structural models of TREK channels and their gating mechanism. *Channels* **5**, 23–33 (2011).
18. Brohawn, S. G., Del Mármol, J. & MacKinnon, R. Crystal structure of the human K2P TRAAK, a lipid- and mechano-sensitive K+ ion channel. *Science* **335**, 436–441 (2012).
19. Jojoa-Cruz, S. *et al.* Cryo-EM structure of the mechanically activated ion channel OSCA1.2. *Elife* **7**, (2018).
20. Buyan, A. *et al.* Piezo1 Forms Specific, Functionally Important Interactions with Phosphoinositides and Cholesterol. *Biophys. J.* **119**, 1683–1697 (2020).
21. Chong, J. *et al.* Modeling of full-length Piezo1 suggests importance of the proximal N-terminus for dome structure. *Biophys. J.* **120**, 1343–1356 (2021).
22. Romero, L. O. *et al.* Dietary fatty acids fine-tune Piezo1 mechanical response. *Nat. Commun.* **10**, (2019).
23. Lee, J. H., Heuser, J. E., Roth, R. & Goodenough, U. Eisosome ultrastructure and evolution in fungi, microalgae, and lichens. *Eukaryot. Cell* **14**, 1017–1042 (2015).
24. Fujita, A., Cheng, J., Tauchi-Sato, K., Takenawa, T. & Fujimoto, T. A distinct pool of phosphatidylinositol 4,5-bisphosphate in caveolae revealed by a nanoscale labeling technique. *Proc. Natl. Acad. Sci. U. S. A.* **106**, 9256–9261 (2009).
25. Fairn, G. D. *et al.* High-resolution mapping reveals topologically distinct cellular pools of phosphatidylserine. *J. Cell Biol.* **194**, 257–275 (2011).
26. Parton, R. G., Kozlov, M. M. & Ariotti, N. Caveolae and lipid sorting: Shaping the cellular response to stress. *J. Cell Biol.* **219**, 1–13 (2020).
27. Anderson, R. H. *et al.* Sterols lower energetic barriers of membrane bending and fission necessary for efficient clathrin-mediated endocytosis. *Cell Rep.* **37**, 110008 (2021).
28. Fu, Y. & Hong, T. T. BIN1 regulates dynamic t-tubule membrane. *Biochim. Biophys. Acta - Mol. Cell Res.* **1863**, 1839–1847 (2016).
29. Mim, C. & Unger, V. M. Membrane curvature and its generation by BAR proteins. *Trends Biochem. Sci.* **37**, 526–533 (2012).
30. Gaus, K., Le Lay, S., Balasubramanian, N. & Schwartz, M. A. Integrin-mediated adhesion regulates membrane order. *J. Cell Biol.* **174**, 725–734 (2006).
31. Seong, J. *et al.* Detection of focal adhesion kinase activation at membrane microdomains by fluorescence resonance energy transfer. *Nat. Commun.* **2**, (2011).
32. Norris, A. & Grant, B. D. Endosomal microdomains: Formation and function. *Curr. Opin. Cell Biol.* **65**, 86–95 (2020).
33. Avalos-Padilla, Y., Georgiev, V. N. & Dimova, R. ESCRT-III induces phase separation in

model membranes prior to budding and causes invagination of the liquid-ordered phase. *Biochim. Biophys. Acta - Biomembr.* **1863**, 183689 (2021).

34. Musse, A. A., Gao, W., Homchaudhuri, L., Boggs, J. M. & Harauz, G. Myelin basic protein as a ‘PI(4,5)P2-modulin’: A new biological function for a major central nervous system protein. *Biochemistry* **47**, 10372–10382 (2008).
35. Ruskamo, S. *et al.* Cryo-EM, X-ray diffraction, and atomistic simulations reveal determinants for the formation of a supramolecular myelin-like proteolipid lattice. *J. Biol. Chem.* **295**, 8692–8705 (2020).
36. Aimon, S. *et al.* Membrane Shape Modulates Transmembrane Protein Distribution. *Dev. Cell* **28**, 212–218 (2014).

Supplementary tables

	Name	Alt. Name	Point group symmetry	Helical rise	Helical twist	Helix Diameter	Reso
**Native-source**	Type 1	7	D2	11.215	54.344	307.9	7.16
**Native-source**	Type 2	0	D1	5.515	-53.522	314.9	3.84
**Native-source**	Type 3	3	D4	21.344	-50.942	322.0	4.28
**Native-source**	Type 4	2	D7	37.299	36.720	325.6	3.72
**Native-source**	Type 5	4	D1	5.173	-136.534	332.6	3.72
**Native-source**	Type 6	5	D1	5.135	49.337	336.2	4.37
**Native-sourcea**	Type 7	6	D2	10.067	-48.121	343.2	3.72
**Native-source**	Type 8	8	D1	4.860	-46.583	353.9	4.02
**Native-source**	Type 9	9	D8	37.570	-57.772	371.6	4.28
**Reconstituted: -PIP2/+sterol**	Type 1	III	D7	36.538	-14.466	334.2	3.89
**Reconstituted: -PIP2/+sterol[a,b]**	Type 2	II	D1	5.044	-136.500	340.9	3.35
**Reconstituted: -PIP2/+sterol**	Type 3	I	D2	9.762	131.906	351.9	3.57
**Reconstituted: +PIP2/-sterol**	Type 1	b	D1	5.420	133.600	323.1	3.91
**Reconstituted: +PIP2/-sterol**	Type 2	a	D4	21.004	39.094	332.0	3.88
**Reconstituted: +PIP2/-sterol**	Type 3	d	D1	5.078	-136.523	345.3	4.65
**Reconstituted: +PIP2/-sterol[a,b,c]**	Type 4	c	D3	14.547	-83.250	351.9	3.86
**Reconstituted: +PIP2/-sterol**	Type 5	e	D1	4.714	-81.141	365.2	4.5
**Reconstituted: +PIP2/+sterol**	Type 1	D	D2	11.142	-137.653	312.1	3.76
**Reconstituted: +PIP2/+sterol**	Type 2	K	D1	5.740	152.300	318.7	6.81
**Reconstituted: +PIP2/+sterol[a,b,c]**	Type 3	A&C	D1	5.408	133.595	325.4	3.61
**Reconstituted: +PIP2/+sterol[a]**	Type 4	FGH	D4	20.968	-140.956	332.0	3.79
**Reconstituted: +PIP2/+sterol**	Type 5	J	D1	5.077	-136.507	343.1	4.52
**Reconstituted: +PIP2/+sterol**	Type 6	E	D1	4.980	-161.053	349.7	3.8
**Reconstituted: +PIP2/+sterol**	Type 7	B	D3	14.548	276.716	356.3	4.03
**Reconstituted: +PIP2/+bromosterol**	Type 1	8b	D2	11.008	42.353	320.9	3.95
**Reconstituted: +PIP2/+bromosterol**	Type 2	9b	D1	5.571	152.247	320.9	4.54
**Reconstituted: +PIP2/+bromosterol[a]**	Type 3	2b	D1	5.338	133.559	330.4	3.85
**Reconstituted: +PIP2/+bromosterol**	Type 4	1b	D4	20.640	219.094	341.8	3.90

Table 1. Symmetry parameters and diameters of helical structures. Helix type (numbered from smallest to largest diameter), alternative name, point group symmetry, helical rise and twist, helix diameter and resolution of helical map. [a]Exemplar helical map used in figures, [b]Helical map used for model refinement, [c]Helical map processed using non-uniform refinement

IUPAC name	Abbreviation	Catalogue #	Purchased from
1,2-dioleoyl-sn-glycero-3-phosphocholine,	DOPC	#850375	Avanti Polar Lipids Inc.
1,2-dioleoyl-sn-glycero-3-phosphoethanoamine	DOPE	#850725	Avanti Polar Lipids Inc.
1,2-dioleoyl-sn-glycero-3-phospho-L-serine	DOPS	#840035	Avanti Polar Lipids Inc.
1-palmitoyl-2-oleoyl-glycero-3-phosphocholine	POPC	#850457	Avanti Polar Lipids Inc.
1-palmitoyl-2-oleoyl-sn-glycero-3-phosphoethanolamine	POPE	#850757	Avanti Polar Lipids Inc.
1-palmitoyl-2-oleoyl-sn-glycero-3-phospho-L-serine	POPS	#840034	Avanti Polar Lipids Inc.
brain L-a-phosphatidylinositol-4,5-biphosphate	PI(4,5)P2	#840046	Avanti Polar Lipids Inc.
cholesterol (ovine)	chol	#700000	Avanti Polar Lipids Inc.
ergosterol, 98%	erg	#117810050	Thermo Scientific
1-oleoyl-2-{6-[4-(dipyrromethenebaron difluoride)butanoyl]amino}hexanoyl-sn-glycero-3-phosphoinositol-4,5-bisphosphate	TopFluor-PI(4,5)P2	#810184	Avanti Polar Lipids Inc.
23-(dipyrrometheneboron difluoride)-24-norcholesterol	TopFluor-cholesterol	#810255	Avanti Polar Lipids Inc.
1-palmitoyl-2-(dipyrrometheneboron difluoride)undecanoyl-sn-glycero-3-phosphocholine	TopFluor-PC	#810281	Avanti Polar Lipids Inc.
1-palmitoyl-2-(dipyrrometheneboron difluoride)undecanoyl-sn-glycero-3-phosphoethanolamine	TopFluor-PE	#810282	Avanti Polar Lipids Inc.
1-palmitoyl-2-(dipyrrometheneboron difluoride)undecanoyl-sn-glycero-3-phospho-L-serine (ammonium salt)	TopFluor-PS	#810283	Avanti Polar Lipids Inc.
ATTO 647N-labelled 1,2-dioleoyl-sn-glycero-3-phosphoethanoamine	Atto 647N-DOPE	#AD 647N-161	ATTO-TEC GmbH

Table 2. Lipids used in this study. IUPAC names, abbreviations used in text, catalogue numbers and supplier.

CryoEM studies

Name	Lipid mixture	molar ratio
-PI(4,5)P2/+sterol	DOPC:DOPE:DOPS:Cholesterol	30:20:20:30 mol%
+PI(4,5)P2/-sterol	DOPC:DOPE:DOPS:brain PI(4,5)P2	50:20:20:10 mol%
+PI(4,5)P2/+sterol	DOPC:DOPE:DOPS:Cholesterol:brain PI(4,5)P_2	35:20:20:15:10 mol%
+PI(4,5)P2/+bromosterol	DOPC:DOPE:DOPS:bromosterol:brain PI(4,5)P_2	35:20:20:15:10 mol%

Lipid sorting coefficient and FRAP studies

Name	Lipid mixture	molar ratio
-PI(4,5)P2/+sterol	DOPC:DOPE:DOPS:cholesterol:TopFluor-cholesterol	30:20:20:29:1 mol%
+1% PI(4,5)P2/+sterol	DOPC:DOPE:DOPS:cholesterol:brain PI(4,5)P2:TopFluor-cholesterol	29:20:20:29:1:1 mol%
+1% PI(4,5)P2/-sterol[a]	DOPC:DOPE:DOPS:TopFluor-PI(4,5)P2	59:20:20:1 mol%
+1% PI(4,5)P2/+sterol[a]	DOPC:DOPE:DOPS:cholesterol:TopFluor-PI(4,5)P2	29:20:20:30:1 mol%
+1% PI(4,5)P2/+sterol[a]	DOPC:DOPE:DOPS:cholesterol:brain P(4,5)P2:TopFluor-PC	28:20:20:30:1:1 mol%
+1% PI(4,5)P2/+sterol[a]	DOPC:DOPE:DOPS:cholesterol:brain P(4,5)P2:TopFluor-PE	29:19:20:30:1:1 mol%
+1% PI(4,5)P2/+sterol[a]	DOPC:DOPE:DOPS:cholesterol:brain P(4,5)P2:TopFluor-PS	29:20:19:30:1:1 mol%
+1% PI(4,5)P2/-sterol	DOPC:DOPE:DOPS:brain P(4,5)P2:TopFluor-PC	58:20:20:1:1 mol%
+1% PI(4,5)P2/-sterol	DOPC:DOPE:DOPS:brain P(4,5)P2:TopFluor-PE	59:19:20:1:1 mol%
+1% PI(4,5)P2/-sterol[a]	DOPC:DOPE:DOPS:brain P(4,5)P2:TopFluor-PS	59:20:19:1:1 mol%
+1% PI(4,5)P2/+ergosterol	DOPC:DOPE:DOPS:ergosterol:TopFluor-PI(4,5)P2	29:20:20:30:1 mol%
+1% PI(4,5)P2/+ergosterol	DOPC:DOPE:DOPS:ergosterol:brain P(4,5)P2:TopFluor-PS	29:20:19:30:1:1 mol%
+1% PI(4,5)P2/+sterol	POPC:POPE:POPS:cholesterol:TopFluor-PI(4,5)P2	29:20:20:30:1 mol%
+1% PI(4,5)P2/+sterol	POPC:POPE:POPS:cholesterol:brain P(4,5)P2:TopFluor-cholesterol	30:20:20:29:1:1 mol%

Table 3. Lipid mixtures used in this study. Mixture name used in text, components of lipid mixtures, and molar ratio (mol %). [a]0.01 mol% of Atto647N DOPE was added to each lipid mixture for lipid sorting experiments

TopFluor-cholesterol

	halftime 1 (s.e.), s	halftime 2 (s.e.), s	mobile fraction (s.e.), %	R-squared
+PIP2, control	5.6 (0.4)	64.1 (8.6)	86.4 (0.5)	0.993
+PIP2, Pil1	0.8 (0.6)	9.3 (0.4)	34.0 (0.1)	0.956
-PIP2, control	5.0 (1.0)	38.5 (3.6)	92.5 (0.9)	0.987
-PIP2, Pil1	0.8 (0.3)	7.4 (0.3)	56.4 (0.1)	0.974
PO-lipids*, control	3.0 (0.4)	16.3 (1.9)	78.9 (0.9)	0.991
PO-lipids*, Pil1	2.25 (0.5)	22.3 (1.2)	40.9 (0.6)	0.995
Pil1 F33A, Y40A, F42A, F50A	0.8 (0.3)	7.4 (0.3)	51.1 (0.1)	0.974

TopFluor-PIP2

	halftime 1 (s.e.), s	halftime 2 (s.e.), s	mobile fraction (s.e.), %	R-squared
+sterol, control	4.8 (0.3)	35.4 (1.2)	60.8 (0.4)	0.998
+sterol, Pil1	4.6 (0.6)	61.9 (4.4)	20.7 (0.6)	0.994
-sterol, control	12.2 (0.3)	-	53.7 (0.2)	0.977
-sterol, Pil1	28.5 (1.0)	-	27.5 (0.3)	0.973
+ergosterol**, control	4.3 (0.5)	48.6 (16.3)	98.8 (11.1)	0.992
+ergosterol**, Pil1	1.6 (0.9)	26.1 (4.1)	19.0 (1.0)	0.953
PO-lipids*, control	2.5 (0.2)	23.4 (0.9)	67.8 (0.6)	0.999
PO-lipids*, Pil1	28.4 (9.4)	-	15.1 (1.3)	0.640

TopFluor-PC

	halftime 1 (s.e.), s	halftime 2 (s.e.), s	mobile fraction (s.e.), %	R-squared
+PIP2, control	11.2 (0.2)	-	74.4 (0.3)	0.989
+PIP2, Pil1	14.4 (0.4)	-	58.7 (0.3)	0.996
-PIP2, control	2.7 (0.3)	18.8 (0.5)	90.7 (0.5)	0.998
-PIP2, Pil1	1.0 (0.1)	13.2 (0.4)	55.9 (0.2)	0.968
-sterol, control	15.4 (0.3)	-	88.5 (0.4)	0.980
-sterol, Pil1	11.5 (0.4)	-	44.3 (0.2)	0.939

TopFluor-PE

	halftime 1 (s.e.), s	halftime 2 (s.e.), s	mobile fraction (s.e.), %	R-squared
+PIP2, control	9.7 (0.1)	-	80.4 (0.2)	0.988
+PIP2, Pil1	9.0 (0.3)	-	49.9 (0.2)	0.926
-PIP2, control	1.9 (0.3)	15.6 (0.3)	81.6 (0.2)	0.995
-PIP2, Pil1	0.8 (0.1)	7.6 (0.5)	54.4 (0.1)	0.951
-sterol, control	13.4 (0.2)	-	80.8 (0.2)	0.990
-sterol, Pil1	5.3 (0.2)	-	54.1 (0.2)	0.925

Table 4. FRAP halftimes in seconds and percentages of mobile fractions. Values are derived from one-phase exponential (one halftime shown) or two-phase exponential (two halftimes shown) equations (see Materials and methods). Standard error of fitted values (s.e.) and the goodness of fits (R-squared) are shown. *PO-lipid mixture = 29mol% POPC : 20mol% POPE : 20mol% POPS : 1mol% PI(4,5)P2 : 30 mol% cholesterol. **ergosterol lipid mixture = 29mol% DOPC : 20mol% DOPE : 20mol% DOPS : 1mol% PI(4,5)P2 : 30 mol% cholesterol.

Name	Genotype	Source
TB50a	*MATa leu2-3,112 ura3-52 rme1 trp1 his3*	Loewith lab
TB50α	*MATα leu2-3,112 ura3-52 rme1 trp1 his3*	PMID:26028537
CG1-37	TB50a *BIT61-TAP::kanMX4*	This study
RL276-2B	TB50α *HIS3*	Loewith lab
MGTY086	TB50α *pil1::KanMX6 lsp1::HphMX4 HIS3*	This study
JK001	TB50α *PIL1-GFPenvy::HIS3MX6 lsp1::HphMX4*	This study
AB001	TB50α *pil1-R43A-GFPenvy::HIS3MX6 lsp1::HphMX4*	This study
AB002	TB50α *pil1-R126A-GFPenvy::HIS3MX6 lsp1::HphMX4*	This study
AB003	TB50α *pil1-K130A/R133A-GFPenvy::HIS3MX6 lsp1::HphMX4*	This study
XZ001	TB50α *pil1-K66A/R70A-GFPenvy::HIS3MX6 lsp1::HphMX4*	This study
XZ002	TB50α *pil1-K66A/R70A/K130A/R133A-GFPenvy::HIS3MX6 lsp1::HphMX4*	This study
XZ003	TB50α *pil1-F33A/Y40A/F42A/F50A-GFPenvy::HIS3MX6 lsp1::HphMX4*	This study
MGTY064	TB50a *pil1-GFPenvy::HIS3MX6 lsp1::HphMX4 NCE102-mScarlet-I::Hph*	This study
JK003	TB50a *pil1-R43A-GFPenvy::HIS3MX6 lsp1::HphMX4 NCE102-mScarlet-I::Hph*	This study
JK004	TB50α *pil1-R126A-GFPenvy::HIS3MX6 lsp1::HphMX4 NCE102-mScarlet-I::Hph*	This study
JK005	TB50a *pil1-K130A/R133A-GFPenvy::HIS3MX6 lsp1::HphMX4 NCE102-mScarlet-I::Hph*	This study
MGTY056	TB50α *pil1-K66A/R70A-GFPenvy::HIS3MX6 lsp1::HphMX4 NCE102-mScarlet-I::Hph*	This study
MGTY062	TB50α *pil1-K66A/R70A/K130A/R133A-GFPenvy::HIS3MX6 lsp1::HphMX4 NCE102-mScarlet-I::Hph*	This study
MGTY061	TB50a *pil1-F33A/Y40A/F42A/F50A-GFPenvy::HIS3MX6 lsp1::HphMX4 NCE102-mScarlet-I::Hph*	This study

Table 5. Yeast strains used in this study. Strain name, genotype, and source.

Source data files

Source data Fig 2.xlsx

Source data Fig 3.xlsx

Source data Fig 4.xlsx

Source data Fig Ext Data 6.xlsx

Source data Fig Ext Data 7.xlsx

Source data Fig Ext Data 8.xlsx

Source data Fig Ext Data 9.xlsx

Source data Fig Ext Data 10.xlsx

Supplementary data

Supplementary Data 1. Mass spectrometry analysis of native-source eisosome preps.
(See .xlsx file**)**

A

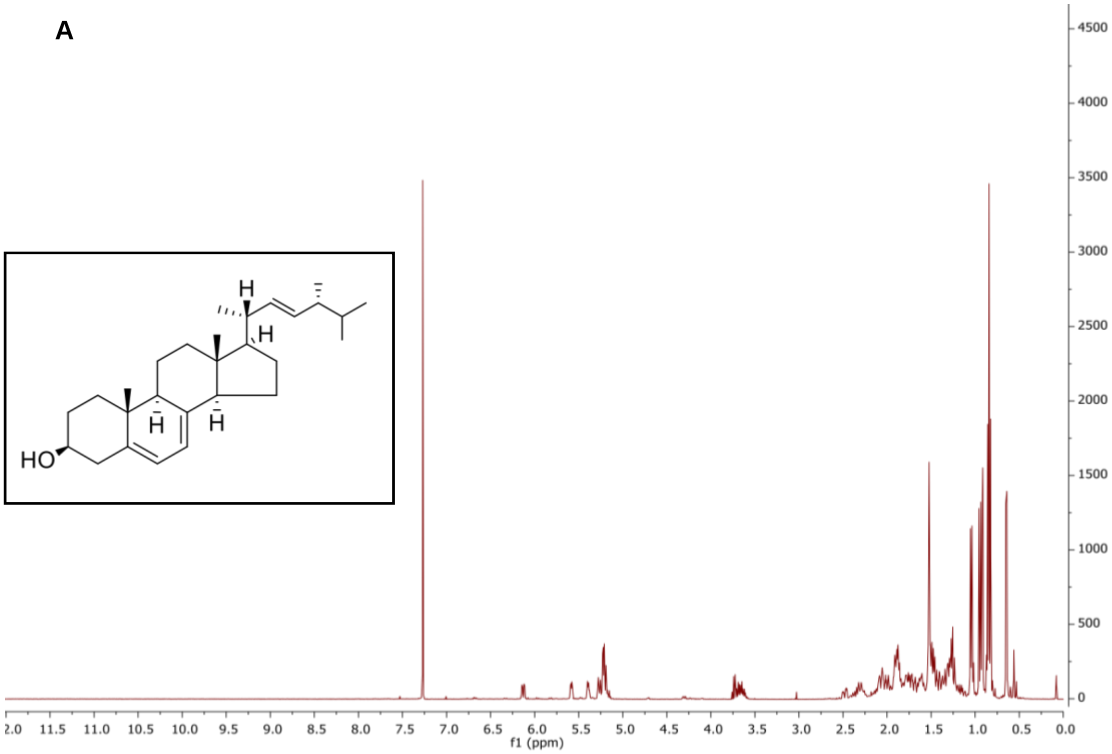

B

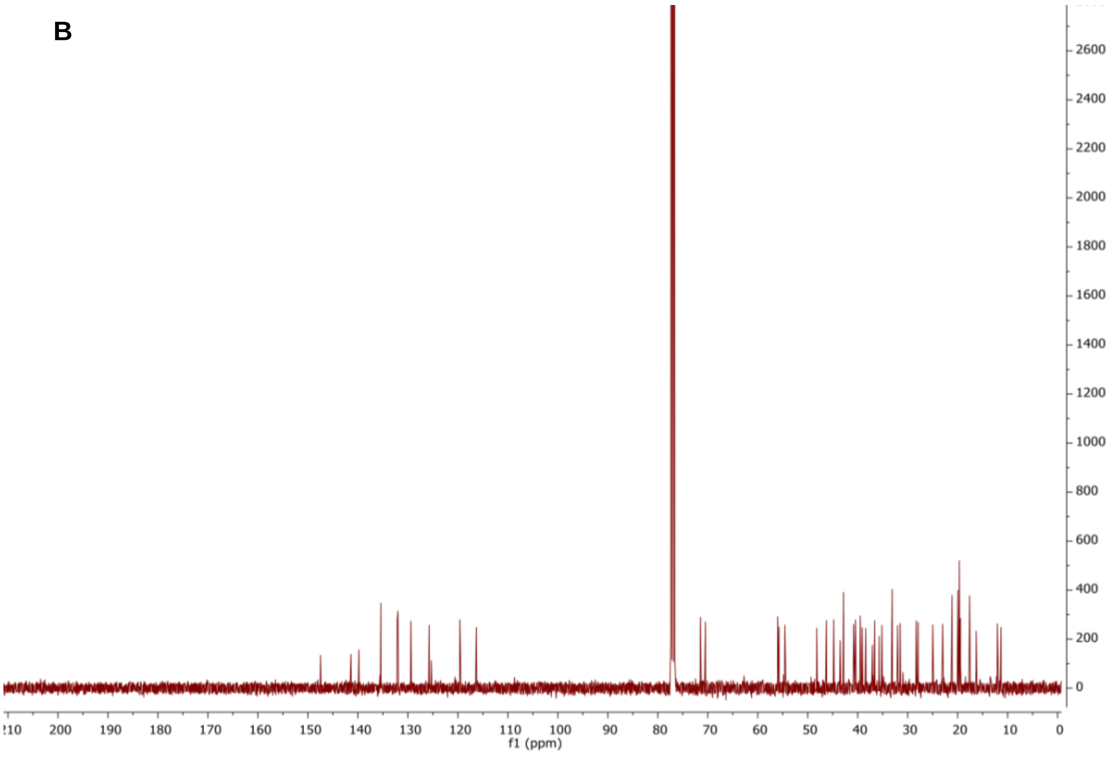

Supplementary Data 2. NMR of ergosterol. A. 1H NMR spectra of ergosterol, inset: structure of ergosterol. **B.** 13C NMR spectra of ergosterol.

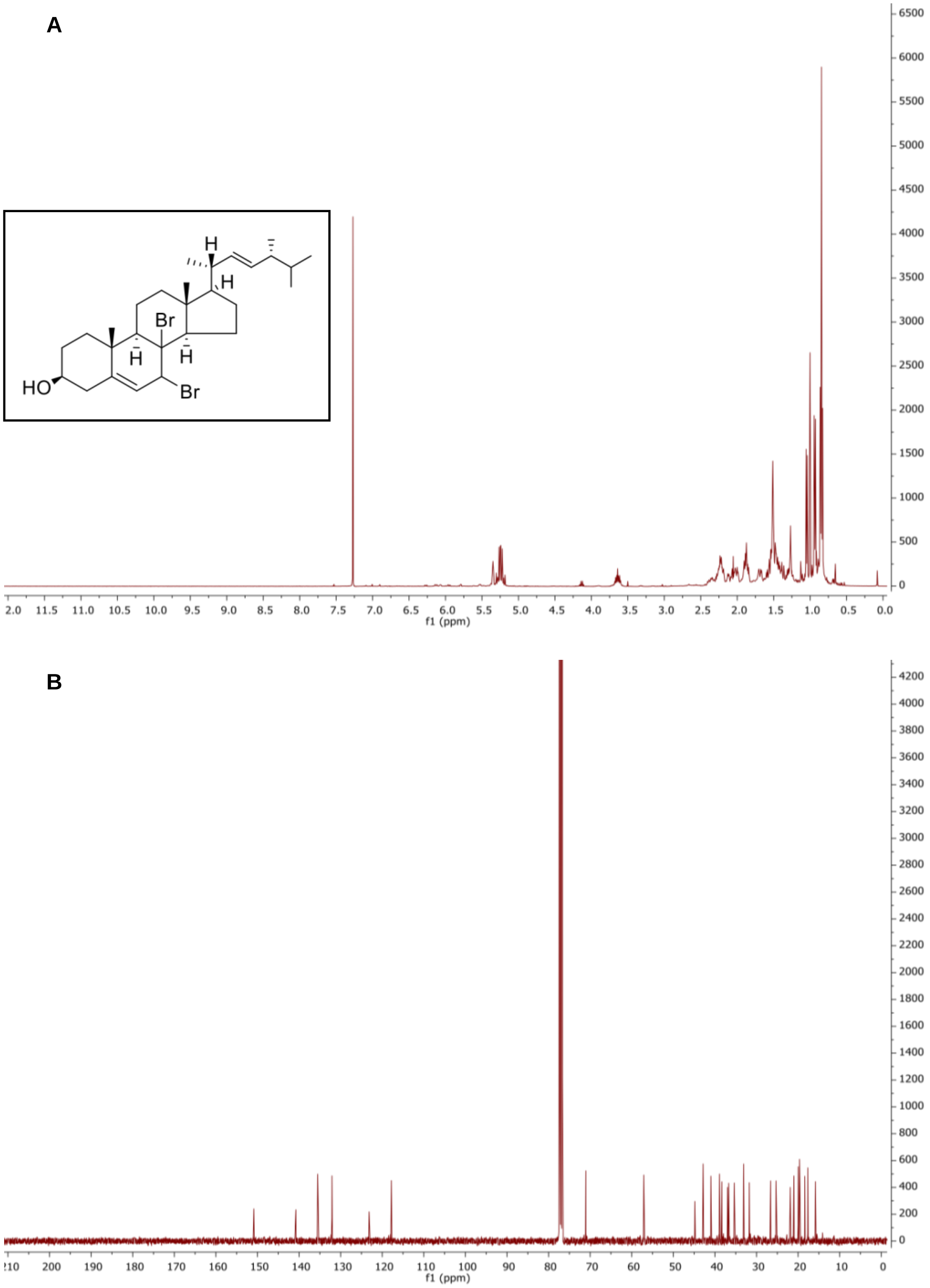

Supplementary Data 3. NMR of brominated ergosterol. A. 1H NMR spectra of brominated ergosterol, inset: Structure of brominated ergosterol. **B.** 13C NMR spectra of brominated ergosterol

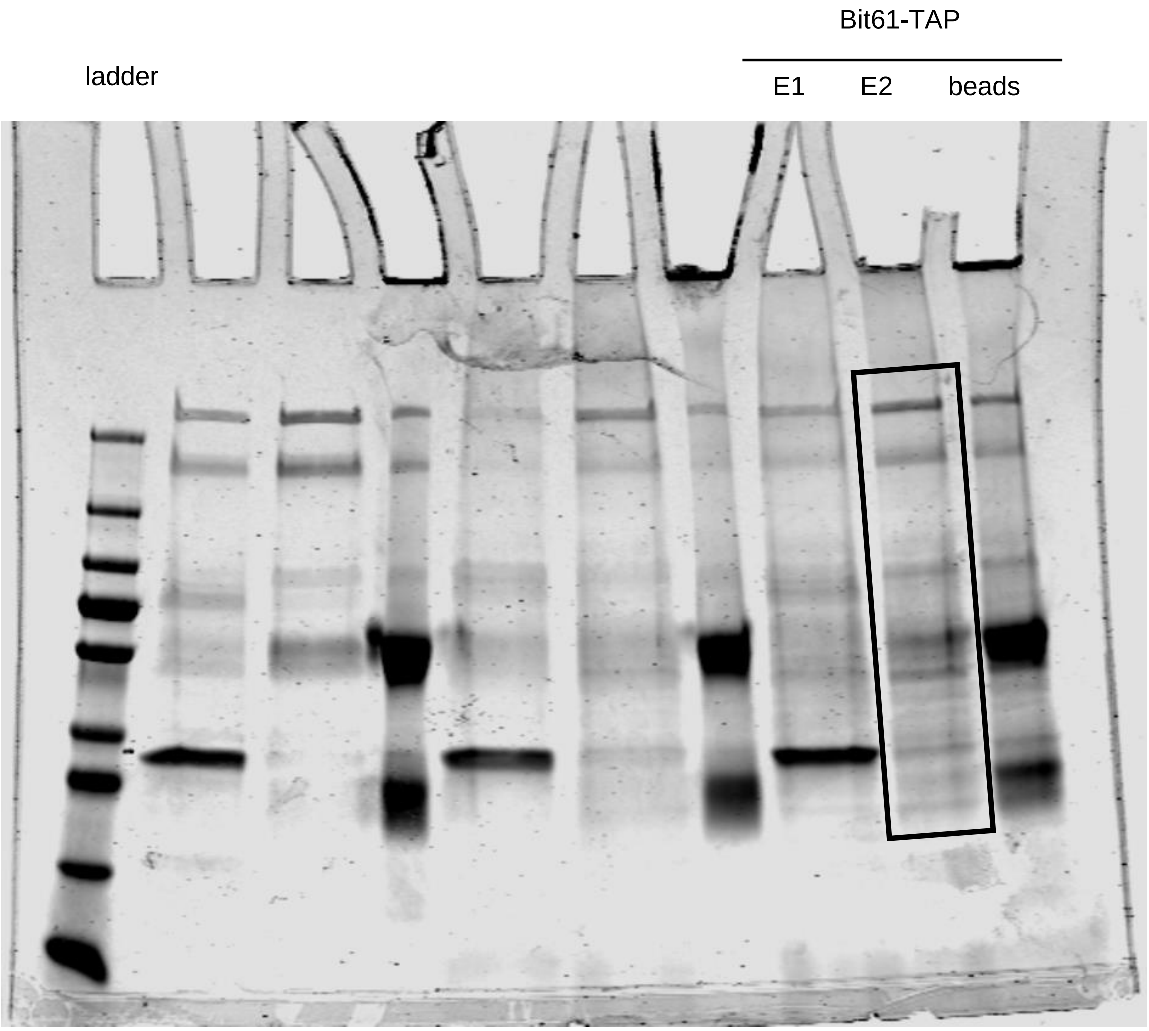

Supplementary Data 4. Source gel image. Coomassie-stained gel loaded with protein from Bit61-TAP purification. Elution 1 (E1), Elution 2 (E2; grids made with this elution), and protein remaining bound to Dynabeads (beads) after elution.
