# Supplementary material for: Cryo-EM architecture of a near-native stretch-sensitive membrane microdomain
Source: Nature. 2024 Jul 24;632(8025):664–71. doi: 10.1038/s41586-024-07720-6 (PMC11324527; doi:10.1038/s41586-024-07720-6)
Supplement: Supplementary file 2 — Reporting Summary [file 41586_2024_7720_MOESM2_ESM.pdf]

nature portfolio

Corresponding author(s): Robbie Loewith

Last updated by author(s): May 30, 2024

Reporting Summary

Nature Portfolio wishes to improve the reproducibility of the work that we publish. This form provides structure for consistency and transparency in reporting. For further information on Nature Portfolio policies, see our Editorial Policies and the Editorial Policy Checklist.

Statistics

For all statistical analyses, confirm that the following items are present in the figure legend, table legend, main text, or Methods section.

n/a	Confirmed	
☐	☒	The exact sample size (*n*) for each experimental group/condition, given as a discrete number and unit of measurement
☐	☒	A statement on whether measurements were taken from distinct samples or whether the same sample was measured repeatedly
☐	☒	The statistical test(s) used AND whether they are one- or two-sided<br>*Only common tests should be described solely by name; describe more complex techniques in the Methods section.*
☐	☒	A description of all covariates tested
☐	☒	A description of any assumptions or corrections, such as tests of normality and adjustment for multiple comparisons
☐	☒	A full description of the statistical parameters including central tendency (e.g. means) or other basic estimates (e.g. regression coefficient) AND variation (e.g. standard deviation) or associated estimates of uncertainty (e.g. confidence intervals)
☐	☒	For null hypothesis testing, the test statistic (e.g. *F*, *t*, *r*) with confidence intervals, effect sizes, degrees of freedom and *P* value noted<br>*Give P values as exact values whenever suitable.*
☒	☐	For Bayesian analysis, information on the choice of priors and Markov chain Monte Carlo settings
☒	☐	For hierarchical and complex designs, identification of the appropriate level for tests and full reporting of outcomes
☒	☐	Estimates of effect sizes (e.g. Cohen's *d*, Pearson's *r*), indicating how they were calculated

Our web collection on statistics for biologists contains articles on many of the points above.

Software and code

Policy information about availability of computer code

Data collection	SerialEM (Nexperion) and EPU 2.14 (ThermoFisher) for EM data collection. BUMPy v1.1 (doi: 10.1021/acs.jctc.8b00765) and GROMACS version 2021.5 (doi.org/10.5281/zenodo.4457591) for CG MD simulations . Slidebook 6.0.22 (Intelligent Imaging) for Spinning disk confocal microscopy experiments and lipid sorting measurements. Visiview v4.4.0.11 (Visitron Systems GmbH) for FRAP experiments. Zeiss Zen 3.3.89.0008, Blue edition for cell imaging. Spectra Manager version 2.14.02 (Jasco) for CD spectrometry.
Data analysis	EM Data: cryoSPARC Live v3.2.2, MotionCor2, Gctf v1.06, RELION v2.1.0 and v3.1.3, Helixplorer-1, cryoSPARC v4.1.2 and v4.4.0 for data processing. Chimera v1.16, ChimeraX v1.5 and Fiji v1.54f, for visualization and figure preparation. Coot 0.8.9.2, phenix.elbow (https://phenix-online.org/documentation/reference/elbow.html), deepEMhancer (https://github.com/rsanchezgarc/deepEMhancer) and PHENIX 1.20-4459 for model building and refinement.<br>Lipid sorting assays: Fiji v1.54f, OriginPro 2022 v9.9.0.225 (OriginLab Corp.) and GraphPad Prism 10.1.1<br>CG MD simulations: GROMACS version 2021.5 (doi.org/10.5281/zenodo.4457591); PyLipID package (doi.org/10.1021/acs.jctc.1c00708); Python version 3; VMD version 1.9.4 (doi: 10.1016/0263-7855(96)00018-5) and Martinize2 (https://github.com/marrink-lab/vermouth-martinize)<br>Mass spec analysis: MaxQuant v1.6.6.0<br>Fluorescence microscopy analysis: Fiji v1.54f, JACoP BIOP version (https://github.com/BIOP/ijp-jacop-b), Cellpose v2.0, and Origin Pro 2022 v9.9.0.225<br>CD spectrometry: Spectra Manager Analysis version 2.15.05 (Jasco) and Origin Pro 2022 v9.9.0.225<br>FRAP analysis: Fiji v1.54f and Origin Pro 2022 v9.9.0.225

For manuscripts utilizing custom algorithms or software that are central to the research but not yet described in published literature, software must be made available to editors and reviewers. We strongly encourage code deposition in a community repository (e.g. GitHub). See the Nature Portfolio guidelines for submitting code & software for further information.

Data

Policy information about availability of data

All manuscripts must include a data availability statement. This statement should provide the following information, where applicable:

- Accession codes, unique identifiers, or web links for publicly available datasets
- A description of any restrictions on data availability
- For clinical datasets or third party data, please ensure that the statement adheres to our policy

Mass spectrometry data were deposited to the ProteomeXchange Consortium via the PRIDE partner repository with the dataset identifier PXD050326.

Sharpened maps used for model refinement and all associated helical maps and deepEMhancer sharpened maps have been deposited in the Electron Microscopy Data Bank (https://www.ebi.ac.uk/emdb/) under the following accession codes: Eisosome native-source (EMD-18307), Pil1 -PIP2/+sterol reconstituted (EMD-18308), Pil1 +PIP2/-sterol reconstituted (EMD-18309), Pil1 +PIP2/+sterol reconstituted (EMD-18310), Pil1 +PIP2/+bromosterol reconstituted (EMD-19822), Eisosome native-source compact (EMD-18311), Eisosome native-source stretched (EMD-18312). Raw micrographs of native-source eisosome samples have been deposited in the Electron Microscopy Public Image Archive (EMPIAR) database under the accession code EMPIAR-12053.

The starting model for building the Pil1 model was acquired from the AlphaFold Protein Structure Database (https://alphafold.ebi.ac.uk/) using the Uniprot accession number P53252 (PIL1_YEAST) and Q12230 (LSP1_YEAST). All models have been deposited in the Protein Data Bank (https://www.rcsb.org/): Pil1 lattice (native-source) (PDB 8QB7), Lsp1 lattice (native-source) (PDB 8QB8), Pil1 lattice (-PIP2/+sterol reconstituted) (PDB 8QB9), Pil1 lattice (+PIP2/-sterol reconstituted) (PDB 8QBB), Pil1 lattice (+PIP2/+sterol reconstituted) (PDB 8QBD), Pil1 lattice compact (native-source) (PDB 8QBE), Pil1 dimer compact with lipid headgroups (native-source) (PDB 8QBF), and Pil1 lattice stretched (native-source) (PDB 8QBG).

Lipid diffusion, lipid sorting and yeast cell biology data are provided at DOI:10.26037/yareta:ubja4xykqzfjbhcfwmg7sgj2x4. Raw gels are provided in Supplementary data. All other data supporting the findings of this study are provided in this manuscript. Source data are provided with this paper.

Research involving human participants, their data, or biological material

Policy information about studies with human participants or human data. See also policy information about sex, gender (identity/presentation), and sexual orientation and race, ethnicity and racism.

Reporting on sex and gender	n/a
Reporting on race, ethnicity, or other socially relevant groupings	n/a
Population characteristics	n/a
Recruitment	n/a
Ethics oversight	n/a

Note that full information on the approval of the study protocol must also be provided in the manuscript.

Field-specific reporting

Please select the one below that is the best fit for your research. If you are not sure, read the appropriate sections before making your selection.

☒ Life sciences ☐ Behavioural & social sciences ☐ Ecological, evolutionary & environmental sciences

For a reference copy of the document with all sections, see nature.com/documents/nr-reporting-summary-flat.pdf

Life sciences study design

All studies must disclose on these points even when the disclosure is negative.

Sample size	EM data: Sizes of cryoEM datasets were determined by microscope availability, efficiency of image collection strategy, and number of tubules visible per micrograph (dependent on sample). For native structures, manual targeting produced a dataset of 2827 movies, sufficiently large to produce 9 helical maps and merged lattice map of good resolution. For reconstituted structures, large (>20,000 movies) datasets were collected, and final particle number after classification was sufficient to achieve good resolution.<br>CG MD simulations: Simulations of 10us were performed in triplicate, thus producing an aggregated time of 30us per system. Sample size was chosen to allow membrane equilibration of multicomponent lipid tubules, as in PMID: 36624348.<br>FRAP assays: Sample sizes were chosen based on previous publications in the field (PMID: 24055060 and PMID: 35858336)<br>Lipid sorting assays: Sample size was chosen based on previous publications on lipid and/or protein sorting experiments using membrane in vitro reconstitutions (PMID:19304798, PMID:20160074, and PMID: 31757972).<br>Cellular microscopy and growth assays: The samples sizes were selected based on previous studies with similar methodologies (PMID: 37902009 and PMID: 29976762).

Data exclusions	EM data: Micrographs and particles of poor quality were excluded as part of the process of EM data analysis to reach good resolution.<br>CG MD simulations: residues >5 angstroms in distance from lipid headgroups were not included in the analysis. Lipid headgroup occupancy lower than 5% was excluded for analysis of DOPS occupancy in +PIP2/-sterol vs +PIP2/+sterol system comparisons and in DOPS vs PIP2 occupancy comparison in the +PIP2/+sterol system.<br>FRAP assays: No data was excluded.<br>Lipid sorting assays: No data was excluded.<br>Cellular microscopy: Segmented cells that where intersected by the image borders, and cells that featured Nce102-Scarlet signal below the fixed threshold (thresholded area = 0) for calculating Manders' co-localisation coefficients were excluded.
Replication	EM data: For each dataset, at least 3 and up to 9 different helical structures were solved from independent sets of particles within the same dataset. Each helical structure within a sample dataset, while varying in diameter and/or helical parameters, exhibited an identical lattice pattern and similar structural features (e.g. lipid headgroups, sterol voids) in regions of interest for our study.<br>CG MD simulations: Three replicas were performed for each system. In addition, as a control, a single replica was performed for the +PIP2/+sterol system composition without the protein. Results were similar across replicas.<br>FRAP assays: repeated three times and in each repetition at least 3 different nanotubes were measured. Results were similar across replicas.<br>Lipid sorting assays: repeated two times and in each repetition at least 8 different nanotubes were measured. Results were similar across replicas.<br>Cellular microscopy: Performed at least three times, on different days, from fresh cultures, yielding similar results. Data from at least two different days was pooled for each strain for overlap analysis. Results were similar across replicas.<br>Yeast growth assays: Performed at least three times, on different days, from fresh cultures, of which at least once from a different streak out from glycerol stock, yielding similar results. Results were similar across replicas.
Randomization	EM data: CryoEM data analysis involves inherent randomization: tubules are randomly oriented within the micrographs, particles are divided into random halfsets and independently processed, and resolution estimates are based on the independently processed half maps.<br>FRAP assays and lipid sorting assays: Randomization was not applied because measuring multiple samples at the same time was not possible. Each sample had to be prepared individually. Samples for each experiment were prepared in a similar manner.<br>Cellular microscopy and growth assays: Yeast strains of known identity were required and used for these assays.
Blinding	EM data: Blinding was not possible because samples of known composition were required.<br>CG MD simulations: Blinding was not done because systems of known composition were required.<br>FRAP assays: Blinding was not possible because the difference between samples without protein and with proteins was significant upon protein binding.<br>Lipid sorting assays: Blinding for group allocation or data analysis was not done since this study relies on studying difference lipid behaviors under Pil1 helical scaffolds<br>Cellular microscopy and growth assays: Blinding was not done as it would not affect the outcome of these experiments.

Reporting for specific materials, systems and methods

We require information from authors about some types of materials, experimental systems and methods used in many studies. Here, indicate whether each material, system or method listed is relevant to your study. If you are not sure if a list item applies to your research, read the appropriate section before selecting a response.

Materials & experimental systems

n/a	Involved in the study	
☒	☐	Antibodies
☒	☐	Eukaryotic cell lines
☒	☐	Palaeontology and archaeology
☒	☐	Animals and other organisms
☒	☐	Clinical data
☒	☐	Dual use research of concern
☒	☐	Plants

Methods

n/a	Involved in the study	
☒	☐	ChIP-seq
☒	☐	Flow cytometry
☒	☐	MRI-based neuroimaging
